# Hypericin alleviates cerebral ischemia/reperfusion injury by modulating endoplasmic reticulum stress

**DOI:** 10.3389/fphar.2025.1723495

**Published:** 2026-01-07

**Authors:** Tingting Li, Chao Wang

**Affiliations:** Department of Neurology, Shanxi Provincial People’s Hospital, Taiyuan, Shanxi, China

**Keywords:** apoptosis, cerebral ischemia/reperfusion, endoplasmic reticulum stress, hypericin, neuroprotection, nimodipine

## Abstract

**Introduction:**

Cerebral ischemia/reperfusion (I/R) injury remains a leading cause of neurological disability and is characterized by oxidative stress, calcium overload, inflammation, and endoplasmic reticulum (ER) stress following reperfusion. Hypericin, a bioactive naphthodianthrone derived from Hypericum perforatum, exhibits antioxidant and anti-apoptotic properties. This study investigated the neuroprotective effects and underlying mechanisms of hypericin in experimental cerebral I/R injury.

**Methods:**

Male Sprague-Dawley rats were subjected to middle cerebral artery occlusion (MCAO) and treated intraperitoneally with hypericin (5, 10, or 20 mg/kg) 30 minutes before reperfusion or nimodipine (10 mg/kg) as a positive control. Neurological severity scores (mNSS), grip strength, rotarod performance, infarct volume, and brain water content were evaluated 24 hours after reperfusion. *In vitro*, murine hippocampal HT22 cells underwent oxygen-glucose deprivation/reoxygenation (OGD/R) and were treated with hypericin (6.25-25 μg/mL). Cell viability (MTT assay), apoptosis (Annexin V/PI flow cytometry), and ER stress-related markers were assessed using qRT-PCR and Western blotting. In silico pharmacokinetic analysis was performed to evaluate blood -brain barrier permeability.

**Results:**

Hypericin significantly reduced cerebral infarct volume by approximately 40%, alleviated brain edema, and improved neurological and motor function compared with untreated I/R animals (p < 0.05). Histopathological and immunohistochemical analyses demonstrated preserved hippocampal structure and reduced caspase-3 activation. In OGD/R-injured HT22 cells, hypericin increased cell viability, reduced apoptotic rates from 30.3% to 10.3%, suppressed ER stress-associated markers (CHOP, GRP78, caspase-12), and normalized the Bax/Bcl-2 ratio. Pharmacokinetic predictions suggested moderate lipophilicity and physicochemical properties compatible with partial blood-brain barrier penetration, particularly under ischemia-induced barrier disruption.

**Discussion:**

These findings demonstrate that hypericin confers significant neuroprotection against cerebral I/R injury by attenuating ER stress-mediated apoptosis and preserving neuronal integrity. Hypericin may represent a promising therapeutic candidate for ischemic stroke.

## 1 Introduction

Cerebral ischemia/reperfusion (I/R) injury poses a substantial clinical challenge, characterized by the restoration of blood flow to ischemic brain tissue, which paradoxically exacerbates neuronal injury through oxidative burst, ionic imbalance, and inflammatory cascades (Kalogeris et al., 2016). This reperfusion phase triggers a complex sequence of biochemical and cellular events, leading to neuronal loss, mitochondrial dysfunction, calcium overload, oxidative stress, excitotoxicity, and neuroinflammation, ultimately impairing cognitive and motor function (Zhang et al., 2024). Compared with ischemia alone, the reperfusion phase introduces a burst of reactive oxygen species (ROS) and triggers inflammatory activation that amplifies neuronal apoptosis (Dirnagl et al., 1999). Unraveling the mechanisms underlying this biphasic injury is crucial for developing effective therapeutic strategies. A critical pathway implicated in I/R-induced neuronal apoptosis involves endoplasmic reticulum (ER) stress and the unfolded protein response (UPR). The ER maintains protein folding, calcium equilibrium, and lipid metabolism (Zheng et al., 2025). Ischemia and subsequent reperfusion disturb ER homeostasis, activating the UPR via the PERK–eIF2α–CHOP and IRE1–XBP1 signaling axes (Liao et al., 2024). While transient UPR activation initially protects cells, persistent ER stress leads to upregulation of CHOP and activation of caspase-12, culminating in apoptosis and neuronal degeneration (Dong et al., 2024; Karademir et al., 2023). Mounting evidence suggests that modulation of ER stress can mitigate ischemic injury and improve neuronal survival, identifying it as a promising therapeutic target (Hetz and Saxena, 2017).

Hypericin, a naturally occurring naphthodianthrone compound extracted from *Hypericum perforatum* (St. John’s Wort), has demonstrated potent antioxidant, anti-inflammatory, and anti-apoptotic activities that may alleviate I/R-induced neurotoxicity (Rychlewski et al., 2023; Yuan et al., 2023). Kraus et al. reported that hypericin attenuates amyloid-β-induced cytotoxicity in microglial cells, reinforcing its neuroprotective potential (Kraus et al., 2007). Similarly, Chang and Wang demonstrated that hypericin reduces glutamate-induced excitotoxicity via MAPK-dependent regulation of synaptic signaling (Chang and Wang, 2010). Furthermore, hypericin improves cognitive performance, suppresses neuroinflammation, and inhibits NLRP3 inflammasome activation in animal models (Jolodar et al., 2021; Zhai et al., 2022). However, whether hypericin exerts neuroprotection in acute ischemic stroke by regulating ER stress–associated apoptosis remains unknown. Based on these observations, we hypothesized that hypericin alleviates neuronal injury following cerebral I/R by modulating ER stress–related apoptotic signaling. To verify this, we employed both *in vivo* (rat middle cerebral artery occlusion, MCAO) and *in vitro* (HT22 cell oxygen–glucose deprivation/reoxygenation, OGD/R) models to investigate the neuroprotective mechanisms of hypericin. Nimodipine, a clinically used calcium-channel blocker with established neuroprotective efficacy, was included as a positive control to validate the experimental system and benchmark the efficacy of hypericin (Tomassoni et al., 2008). This study aims to elucidate whether modulation of ER stress by hypericin contributes to its neuroprotective action and to explore its translational potential as a novel therapeutic agent for ischemic stroke.

## 2 Materials and methods

### 2.1 Animal subjects

Male Sprague–Dawley rats (6–8 weeks old, 200–250 g) were obtained from Shanghai SLAC Laboratory Animal Co., Ltd. (Shanghai, China) and housed under standard laboratory conditions (22 °C ± 2 °C, 12 h light/dark cycle) with free access to food and water. The sample size (n = 8 per group) was determined based on previous MCAO studies demonstrating that 6–10 animals per group are adequate to detect statistically significant neuroprotective effects while adhering to ethical standards (Wang et al., 2025; Peng et al., 2024). All experimental procedures complied with institutional and national guidelines for animal welfare and were approved by the Institutional Animal Care and Use Committee (IACUC) of Shanxi Provincial People’s Hospital (Approval No: PPH601/23). Experiments were reported following the ARRIVE 2.0 guidelines. At the conclusion of the experimental procedures, all rats were humanely euthanized with an overdose of sodium pentobarbital (150 mg/kg, intraperitoneally) in strict accordance with the CPCSEA and AVMA 2020 Guidelines for the Euthanasia of Animals.

### 2.2 Induction of cerebral ischemia/reperfusion injury

Focal cerebral ischemia was induced by the intraluminal middle cerebral artery occlusion (MCAO) method (Longa et al., 1989). Under anesthesia (2% isoflurane in 70% N_2_O and 30% O_2_), a silicone-coated 4–0 nylon filament (Beijing Cinontech Co., China) was introduced into the internal carotid artery to occlude the middle cerebral artery. After 90 min of occlusion, the filament was gently withdrawn to initiate reperfusion for 24 h. In the sham group, the filament was inserted but not advanced into the MCA. Body temperature was maintained at 37 °C ± 0.5 °C throughout the procedure using a feedback-controlled heating pad.

### 2.3 Drug administration and experimental groups

Animals were randomly divided into six groups (n = 8 per group): Sham, MCAO, MCAO + Hypericin (5 mg/kg), MCAO + Hypericin (10 mg/kg), MCAO + Hypericin (20 mg/kg), and MCAO + Nimodipine (10 mg/kg). Hypericin (≥98% purity, Sigma-Aldrich, United States) was dissolved in DMSO and administered intraperitoneally (0.5 mL per rat) 30 min before reperfusion. All groups including sham received equivalent DMSO volumes to eliminate solvent-related effects. Nimodipine (Sigma-Aldrich, United States) was prepared and administered in the same vehicle at 10 mg/kg. The dose selection for hypericin (5–20 mg/kg) was based on previous reports of neuroprotective efficacy in rodent models and pilot titration within this study demonstrating behavioral and histological improvement without hepatotoxicity (Supplementary Figure S1) (Kraus et al., 2007; Chang and Wang, 2010; Oliveira et al., 2016; Lv et al., 2019).

### 2.4 Neurological deficit evaluation

Neurological function was evaluated 24 h after reperfusion using the modified Neurological Severity Score (mNSS) (Dong et al., 2024). This composite 18-point scale assesses motor, sensory, reflex, and balance functions; higher scores indicate more severe deficits.

### 2.5 Assessment of brain water content

Brain edema was assessed by the wet–dry weight method (Ma et al., 2019). After 24 h of reperfusion, brains were rapidly removed and weighed (wet weight), dried at 100 °C for 24 h, and reweighed (dry weight).

$$\text{Water content }\left(\%\right)=\left(Wet-Dry\right)/Wet\times 100$$

### 2.6 Infarct volume determination

Brains were cut into 2 mm-thick coronal sections and stained with 2% 2,3,5-triphenyltetrazolium chloride (TTC) at 37 °C for 30 min (Li et al., 2015). Images were analyzed using ImageJ software (NIH, United States), and infarct volume was expressed as a percentage of the contralateral hemisphere after edema correction.

### 2.7 Histopathological and toxicity evaluation

Hippocampal tissues were fixed in 4% paraformaldehyde for 24 h, dehydrated through graded ethanol, embedded in paraffin, and sectioned (5 µm). Sections were stained with hematoxylin for 5 min and eosin for 2 min, then observed under a light microscope (Nikon Eclipse Ci, Japan) at ×100 magnification. Liver sections were similarly processed and evaluated for hepatotoxicity. Normal hepatic architecture and preserved nuclear morphology was observed, confirming the absence of hypericin-induced toxicity.

### 2.8 Grip strength measurement

Motor strength was assessed with a Grip Strength Meter (Columbus Instruments, United States). Each rat was allowed to grasp a bar, and the maximum force (g) before release was recorded. Three trials per rat were performed, and the mean was used.

### 2.9 Foot misplacement and tail error tests

Sensorimotor coordination was assessed using a grid-walking task. Rats were placed on an elevated wire grid, and both front leg errors and tail slips were recorded for 1 minute. A tail slip was defined as failure to place or maintain the tail properly on the grid.

### 2.10 Rotarod test

The Rotarod apparatus (Ugo Basile, Italy) was used to evaluate motor coordination and balance. Rats were pretrained to stay on the rod at 10 rpm for 60 s. After reperfusion, testing was performed at accelerating speeds (4–40 rpm over 5 min). Each rat underwent three trials, and mean latency to fall was recorded.

### 2.11 Immunohistochemistry

Brain sections (4–5 µm) were deparaffinized, rehydrated, and subjected to antigen retrieval (citrate buffer, pH 6.0). After blocking with 5% goat serum, sections were incubated overnight at 4 °C with anti-caspase-3 antibody (Abcam, ab4051, 1:200), followed by HRP-conjugated secondary antibody. Immunoreactivity was visualized using DAB substrate, counterstained with hematoxylin, and imaged at 50 µm scale under a light microscope (Ramos-Vara, 2005).

### 2.12 *In vitro* oxygen–glucose deprivation/reoxygenation (OGD/R)

HT22 murine hippocampal cells (ATCC, United States) were cultured in DMEM (Gibco, United States) supplemented with 10% FBS (Gibco, United States). OGD was induced for 3 h in glucose-free DMEM within a hypoxic chamber (1% O_2_, 5% CO_2_, 94% N_2_). Reoxygenation was achieved by replacing with normal DMEM and incubating under normoxia for 12 h (Yu et al., 2021). Groups included: Control, OGD/R, OGD/R + Hypericin (6.25, 12.5, and 25 μg/mL), and OGD/R + Nimodipine (2.5 μg/mL). DMSO was used in all groups, including the control group, to ensure solvent consistency across treatments. (Note: *in vitro* doses are expressed in µg/mL for comparability with literature (Akkuş et al., 2022; Delaey et al., 2001); these are not directly equivalent to *in vivo* mg/kg doses).

### 2.13 MTT assay and phase-contrast microscopy

HT22 cells (1 × 10^4^ per well) were seeded in 96-well plates. After OGD/R, cells were incubated with 20 µL MTT solution (5 mg/mL, Sigma-Aldrich, United States) for 4 h at 37 °C (Mosmann, 1983). Formazan crystals were dissolved in 150 µL dimethyl sulfoxide (DMSO), and absorbance was read at 570 nm using a microplate reader (Bio-Rad, United States).

### 2.14 Annexin V/PI apoptosis assay

Apoptotic cells were quantified using the Annexin V-FITC/PI kit (BD Biosciences, United States). Briefly, 1 × 10^6^ cells were stained with Annexin V-FITC and PI for 15 min and analyzed on a BD FACSCalibur flow cytometer. Early and late apoptotic cells were expressed as percentages of total cells.

### 2.15 Quantitative real-time PCR (qRT-PCR)

Total RNA was extracted from both ischemic brain tissues and HT22 cells using Trizol reagent (Invitrogen, United States). Reverse transcription was performed using PrimeScript RT reagent kit (Takara, Japan). qRT-PCR was carried out with SYBR Green Master Mix (Applied Biosystems, United States) on an ABI 7500 system. Target genes included Bax, Bcl-2, Caspase-3, Caspase-9, Caspase-12, GRP78, and CHOP, with GAPDH as the internal control. Expression was calculated using the 2^−ΔΔCt^ method. Primer sequences are listed in Supplementary Table S1.

### 2.16 Western blotting

Protein levels of CHOP, GRP78, Caspase-12, and Cleaved Caspase-3 were analyzed in HT22 cells after OGD/R treatment. Cells were lysed in RIPA buffer with protease inhibitors, and equal protein amounts (30 µg) were separated by SDS-PAGE and transferred to PVDF membranes. After blocking with 5% BSA, membranes were incubated overnight at 4 °C with primary antibodies against CHOP, GRP78, Caspase-12, Cleaved Caspase-3, and β-actin (1:1000; Abcam), followed by HRP-conjugated secondary antibodies (1:5000; CST). Bands were visualized using an ECL kit (Bio-Rad).

### 2.17 Statistical analysis

All experimental data were expressed as mean ± standard deviation (SD). Prior to analysis, data distributions were assessed for normality using the Shapiro–Wilk test. For datasets exhibiting normal distribution, comparisons among multiple groups were performed using one-way analysis of variance (ANOVA) followed by Dunnett’s *post hoc* test for pairwise comparisons. Non-parametric data, including neurological deficit scores (mNSS), foot misplacement, and tail error tests, were analyzed using the Kruskal–Wallis test with Dunn’s *post hoc* correction. When only two groups were compared, the Student’s *t*-test was applied. Exact *p*-values were reported wherever possible, and differences were considered statistically significant at *p* < 0.05. All statistical analyses were conducted using GraphPad Prism 8.0 software (GraphPad Software, United States) to ensure reproducibility and transparency in data evaluation.

### 2.18 *In silico* ADME and BBB permeability prediction

The physicochemical and pharmacokinetic properties of hypericin were evaluated using the SwissADME online tool (https://www.swissadme.ch). Canonical SMILES string of hypericin was entered to predict lipophilicity (Log P), solubility, gastrointestinal (GI) absorption, blood–brain barrier (BBB) permeability, bioavailability score, and Lipinski rule compliance.

## 3 Results

### 3.1 Effect of hypericin on motor function and coordination

**FIGURE 1:**
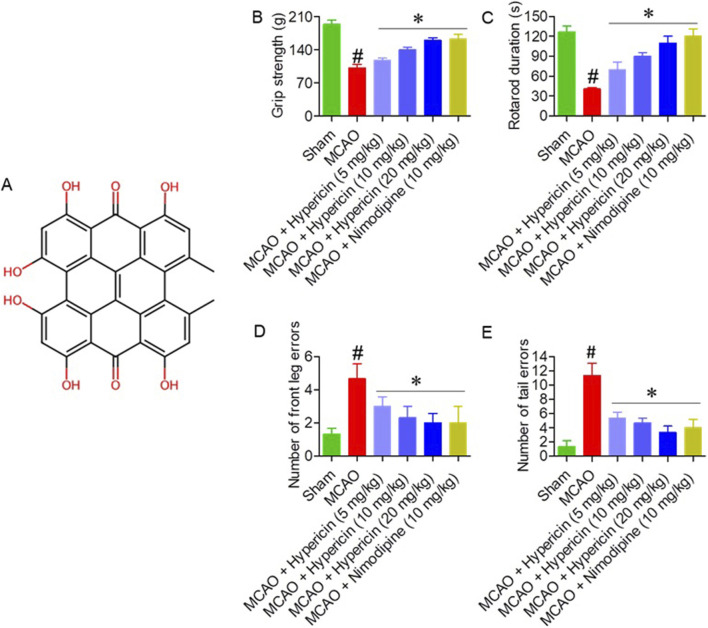
Hypericin improves motor performance in MCAO rats. **(A)** Chemical structure of hypericin. Effects on **(B)** grip strength, **(C)** rotarod latency, **(D)** foot-misplacement errors, and **(E)** tail-placement errors across experimental groups (n = 8 rats per group). Data are presented as mean ± SD. Behavioral scores (foot-misplacement, and tail-placement error tests) were analyzed using the Kruskal–Wallis test followed by Dunn’s *post hoc* test; continuous variables (grip strength and rotarod latency) were analyzed using one-way ANOVA with Dunnett’s *post hoc* test. #P < 0.05 vs. Sham (Student’s t-test); *P < 0.05 vs. MCAO (one-way ANOVA with Dunnett’s *post hoc* test).

The effects of hypericin (Figure 1A) on motor function were evaluated across all experimental groups (n = 8 per group). Grip strength markedly increased in the MCAO + Hypericin (20 mg/kg) group (160 ± 11 g) compared to the MCAO group (101 ± 8 g, p < 0.01) (Figure 1B). Rotarod latency improved from 42 ± 5 s in the MCAO group to 110 ± 7 s after 20 mg/kg hypericin treatment (p < 0.01) (Figure 1C). In both the Foot Misplacement and Tail Error tests, hypericin-treated rats exhibited a 45%–55% reduction in foot slips and tail errors, reflecting improved sensorimotor coordination (p < 0.05) (Figures 1D,E).

### 3.2 Impact of hypericin on neurological function, brain water content, and infarct volume

**FIGURE 2:**
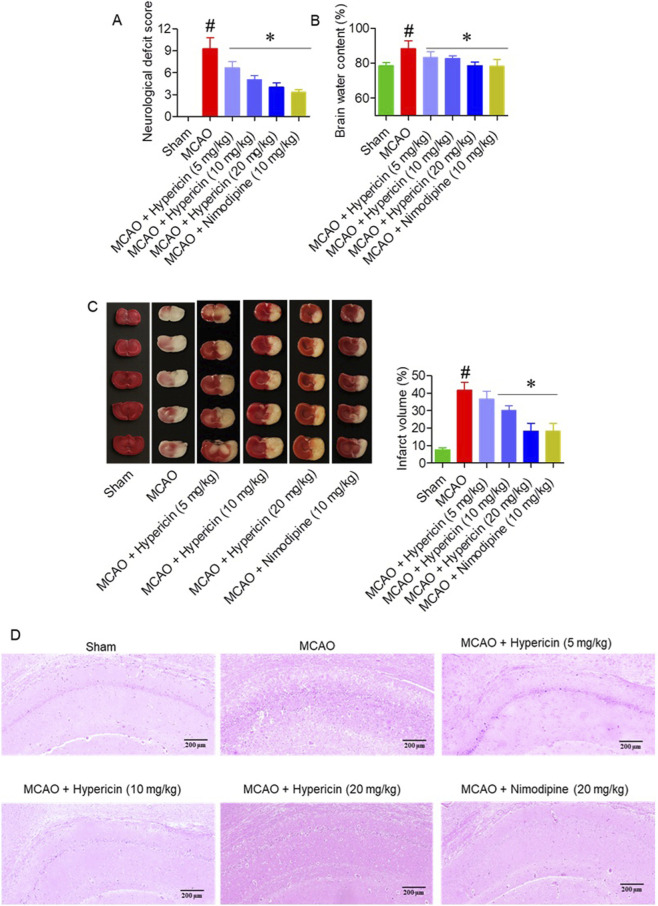
Hypericin alleviates cerebral ischemia/reperfusion (I/R) injury and brain edema. **(A)** Neurological severity scores (mNSS), **(B)** brain-water content, **(C)** infarct volume with representative TTC-stained coronal sections, and **(D)** hematoxylin and eosin, (H&E) staining of hippocampal CA1 neurons (n = 8 rats per group). Data are mean ± SD. The Kruskal–Wallis test with Dunn’s correction was used for mNSS, while continuous variables were analyzed by one-way ANOVA with Dunnett’s *post hoc* test. #P < 0.05 vs. Sham; *P < 0.05 vs. MCAO. **(D)** H&E staining, hippocampal CA1 region; magnification ×100; scale bar = 200 µm.

Hypericin significantly improved neurological recovery, as indicated by lower modified Neurological Severity Scores (mNSS) in all treatment groups compared with MCAO (p < 0.05) (Figure 2A). The MCAO group exhibited elevated brain water content (88.3% ± 2.8%), while hypericin administration reduced edema to 83.3% ± 1.9% (5 mg/kg), 82.6% ± 1.4% (10 mg/kg), and 78.6% ± 1.5% (20 mg/kg) (p < 0.01) (Figure 2B). TTC staining confirmed that hypericin (20 mg/kg) reduced infarct volume by approximately 40% compared to the MCAO group (Figure 2C). Histopathological analysis of hippocampal CA1 neurons showed preserved pyramidal architecture, reduced cytoplasmic vacuolation, and intact nuclei in hypericin-treated rats, whereas MCAO animals exhibited marked neuronal disorganization and loss (Figure 2D). Histopathological analysis of liver tissues showed normal hepatic architecture and preserved nuclear morphology across all groups, confirming the absence of hypericin-induced toxicity (Supplementary Figure S1).

### 3.3 Influence of hypericin on apoptosis

**FIGURE 3:**
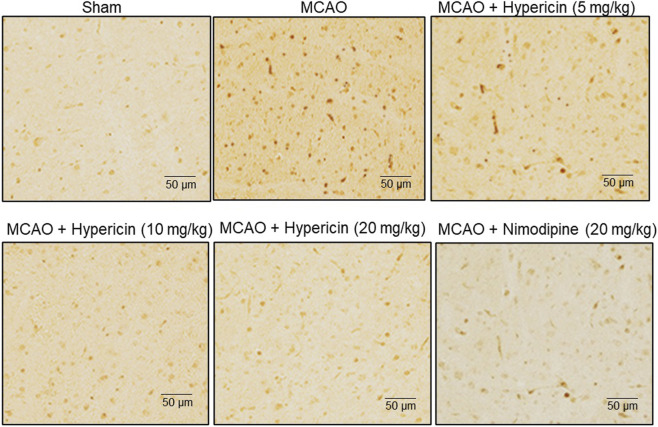
Hypericin reduces neuronal apoptosis in MCAO rat brain tissues. Representative immunohistochemical (IHC) staining of caspase-3 expression in hippocampal CA1 neurons. Low- and high-power fields are shown (scale bar = 50 µm). Quantitative analysis was based on n = 8 rats per group, with experiments performed in triplicate. #P < 0.05 vs. Sham (Student’s t-test); *P < 0.05 vs. MCAO (one-way ANOVA with Dunnett’s *post hoc* test).

Immunohistochemistry for caspase-3 revealed elevated expression in MCAO rats, which was markedly reduced following hypericin treatment, especially at 20 mg/kg (Figure 3). This supports the anti-apoptotic effect of hypericin *in vivo*.

### 3.4 Neuroprotective effects of hypericin against OGD/R-induced HT22 cell damage

**FIGURE 4:**
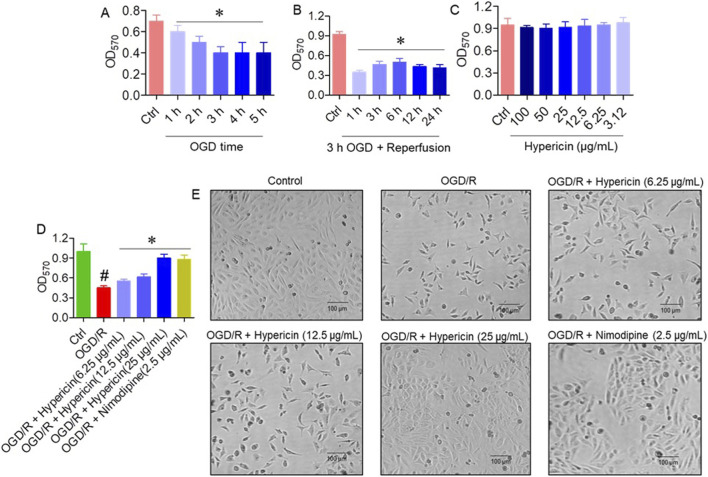
Hypericin protects HT22 cells from oxygen–glucose deprivation/reoxygenation (OGD/R)-induced injury. **(A)** Cell viability following different durations of OGD, **(B)** cell viability after 3 h OGD plus varying reoxygenation periods, **(C)** effect of hypericin concentrations on viability, **(D)** cell viability across experimental groups, and **(E)** representative phase-contrast images. Data are mean ± SD (n = 3 independent experiments). #P < 0.05 vs. Control (Student’s t-test); *P < 0.05 vs. OGD/R (one-way ANOVA with Dunnett’s *post hoc* test).

In the OGD/R model, HT22 cells were exposed to 1–5 h of OGD. Cell viability progressively declined after 3 h of OGD (Figure 4A), and therefore 3 h OGD followed by reoxygenation was used for subsequent experiments. Following reperfusion (1–24 h), viability significantly decreased after 12 h compared to earlier time points (Figure 4B). Different concentrations of hypericin were tested, and 6.25 μg/mL demonstrated optimal protective activity (Figure 4C). Treatment with hypericin significantly improved viability after OGD/R. The OGD/R + Hypericin (6.25, 12.5, and 25 μg/mL) groups showed absorbance values of 0.45 ± 0.03, 0.55 ± 0.02, and 0.70 ± 0.05, respectively, compared with 0.20 ± 0.05 in the OGD/R group (p < 0.05, ANOVA with Dunnett’s *post hoc* test) (Figure 4D). Phase contrast microscopy confirmed that hypericin preserved normal morphology, with fewer apoptotic features (shrinkage, blebbing) in treated groups (Figure 4E).

### 3.5 Effect of hypericin on apoptosis of HT22 cells

**FIGURE 5:**
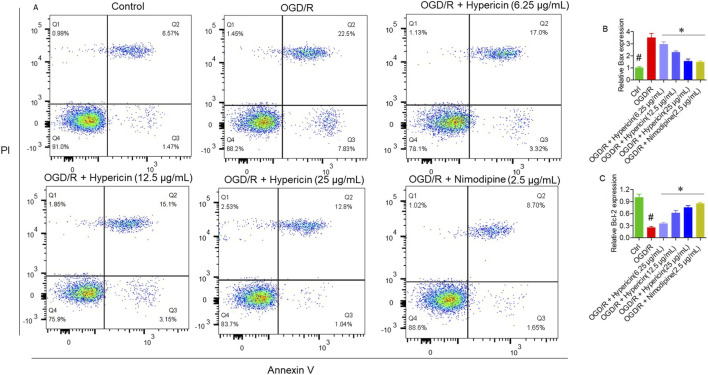
Hypericin suppresses OGD/R-induced apoptosis in HT22 cells. **(A)** Annexin V/PI flow-cytometry plots showing apoptotic-cell percentages, **(B)** relative mRNA expression of Bax, and **(C)** relative mRNA expression of Bcl-2. Data are mean ± SD (n = 3 independent experiments). #P < 0.05 vs. Control (Student’s t-test); *P < 0.05 vs. OGD/R (one-way ANOVA with Dunnett’s *post hoc* test).

Annexin V/PI flow cytometry confirmed a significant reduction in apoptosis, with 10.35% apoptotic cells in the OGD/R + Hypericin (25 μg/mL) group versus 30.33% in the OGD/R group (p < 0.01) (Figure 5A). qRT-PCR analysis showed that hypericin downregulated pro-apoptotic Bax and upregulated anti-apoptotic Bcl-2 expression (p < 0.05), further supporting its cytoprotective effect (Figures 5B,C).

### 3.6 Modulation of endoplasmic reticulum stress by hypericin

**FIGURE 6:**
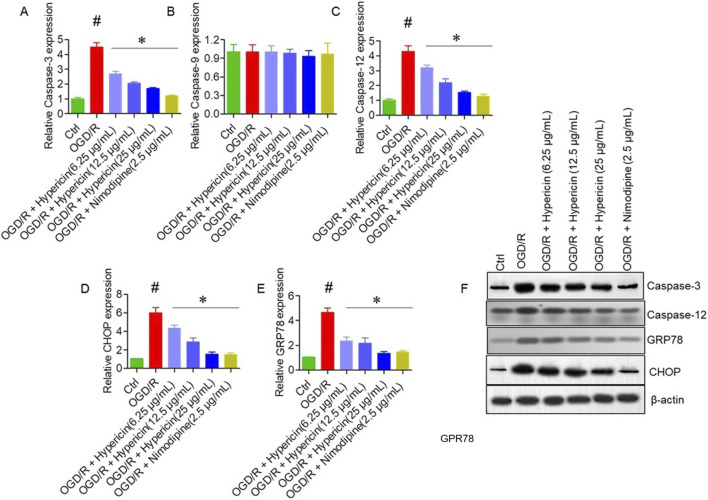
Hypericin modulates endoplasmic reticulum (ER) stress–related apoptotic markers in HT22 cells. Relative mRNA expression of **(A)** caspase-3, **(B)** caspase-9, **(C)** caspase-12, **(D)** CHOP (CCAAT/enhancer-binding protein homologous protein), and **(E)** GRP78 (glucose-regulated protein 78). **(F)** Representative Western blots showing caspase-3, caspase-12, CHOP, and GRP78 expression across groups. Data are mean ± SD (n = 3 independent experiments). #P < 0.05 vs. Control (Student’s t-test); *P < 0.05 vs. OGD/R (one-way ANOVA with Dunnett’s *post hoc* test).

qRT-PCR results demonstrated that OGD/R significantly elevated mRNA levels of Caspase-3, Caspase-12, CHOP, and GRP78, but not Caspase-9. Hypericin treatment attenuated CHOP, GRP78, and Caspase-12 expression in a dose-dependent manner (p < 0.05), indicating suppression of ER stress–mediated apoptosis (Figures 6A–E). Western blotting further validated these findings (Figure 6F). Compared with the OGD/R group, hypericin significantly downregulated CHOP, GRP78, Caspase-12, and caspase-3 protein levels, with 25 μg/mL concentration producing the greatest reduction (p < 0.01) confirming that hypericin mitigates ER stress–associated apoptotic signaling in HT22 cells.

### 3.7 *In silico* prediction of ADME properties and BBB permeability

The pharmacokinetic characteristics of hypericin were analyzed using the SwissADME platform (Supplementary Table S2). Hypericin demonstrated a molecular weight of 504.44 Da, WLOGP 5.32, and a topological polar surface area (TPSA) of 155 Å^2^, indicating moderate lipophilicity and limited polarity that support interaction with biological membranes. Although its physicochemical profile suggests restricted passive diffusion across an intact blood–brain barrier, transient permeability changes during ischemia/reperfusion may facilitate its cerebral entry. The predicted bioavailability score (0.17) and compliance with major drug-likeness criteria further support its potential as a CNS-active compound. Together, these *in silico* predictions complement the experimental findings and reinforce that hypericin can attain pharmacologically relevant brain exposure under ischemic conditions, contributing to its observed neuroprotective efficacy.

## 4 Discussion

Cerebral ischemia/reperfusion (I/R) injury remains a major clinical problem, often resulting in long-term neurological disability and high morbidity. The need for effective neuroprotective strategies is underscored by the multiple pathophysiological processes involved, including excitotoxicity, oxidative stress, inflammation, and apoptosis (Jurcau and Simion, 2021; Lee et al., 2018). In this context, our study provides comprehensive evidence that hypericin exerts potent neuroprotective effects in both *in vivo* and *in vitro* models of I/R injury. The combined use of MCAO and OGD/R models strengthens the translational value of our findings, as these systems simulate both the vascular and cellular dimensions of reperfusion injury. Consistent with previous reports (Yuan et al., 2023; Kraus et al., 2007; Chang and Wang, 2010; Jolodar et al., 2021), hypericin treatment significantly improved neurological performance, reduced cerebral infarction, and attenuated cerebral edema, indicating that it effectively mitigates reperfusion-induced damage. Behavioral recovery, reflected in enhanced grip strength, rotarod balance, and reduced mNSS scores, further supports its functional efficacy. These findings align with studies demonstrating that *H. perforatum* extracts improve motor and cognitive outcomes in ischemic models through antioxidative and anti-inflammatory mechanisms (Shoaib et al., 2023; Crupi et al., 2011). Interestingly, hypericin achieved neuroprotection comparable to nimodipine, a clinically used calcium channel blocker (Tomassoni et al., 2008; Chen et al., 1996; Shen et al., 2017), suggesting that its actions may converge on similar pathways of neuronal preservation, albeit through broader mechanisms involving oxidative stress modulation and ER homeostasis.

At the cellular level, hypericin markedly reduced neuronal apoptosis, as demonstrated by decreased caspase-3 immunoreactivity in MCAO rats and reduced Annexin V/PI positivity in OGD/R-treated HT22 cells. These effects were accompanied by downregulation of pro-apoptotic Bax and upregulation of anti-apoptotic Bcl-2, thereby restoring the Bax/Bcl-2 ratio—a critical determinant of neuronal survival following ischemic insult (Schäbitz et al., 2000; Shiraishi et al., 2006; Ardic et al., 2019). Mechanistically, our study provides evidence that hypericin mitigates ER stress–mediated apoptosis, as indicated by decreased expression of CHOP, GRP78, and caspase-12. CHOP and GRP78 serve as master regulators of the unfolded protein response, while caspase-12 mediates ER-specific apoptotic signaling (Matsuo et al., 2013; Costantini et al., 2002). By downregulating these markers, hypericin likely restores ER homeostasis and interrupts ER-dependent apoptotic cascades.

However, whether hypericin directly interferes with the PERK–eIF2α–CHOP signaling axis or indirectly reduces ER stress via its antioxidative activity remains to be clarified. It is plausible that hypericin acts both as a direct modulator of ER stress sensors and an indirect stabilizer through suppression of ROS and calcium overload, which are upstream triggers of ER dysfunction. Further validation through phosphorylation assays of PERK and eIF2α or antioxidant inhibition studies would help delineate its precise molecular target within the ER stress pathway. Notably, caspase-9, a mitochondrial apoptosis marker (Li et al., 2000), remained unaltered, suggesting that hypericin preferentially targets ER stress–related apoptosis rather than mitochondrial pathways. This selective modulation differentiates it from agents such as cyclosporine A, which act mainly by stabilizing mitochondrial membranes (Paul Kamdem et al., 2016).

*In silico* pharmacokinetic predictions support plausibility of hypericin as a CNS-active compound. Although physicochemical parameters suggest limited passive BBB diffusion under normal conditions, its moderate lipophilicity (WLOGP 5.32) and TPSA of 155 Å^2^ indicate a potential for partial penetration, particularly during ischemic episodes when BBB permeability is transiently increased. Such conditions have been shown to facilitate CNS entry of lipophilic phytochemicals (Qi et al., 2024; Sun et al., 2025). These predictions, combined with the significant *in vivo* efficacy observed, suggest that hypericin can reach pharmacologically relevant cerebral concentrations during reperfusion. Toxicological analysis further confirmed safety at therapeutic doses, as H&E-stained liver sections revealed intact hepatic, preserved sinusoidal spaces, and normal cytoplasmic and nuclear morphology. This indicates that hypericin, even at 20 mg/kg, is non-hepatotoxic under the tested conditions, consistent with reports of its favorable safety margin in other preclinical models (Li et al., 2012).

Despite these encouraging results, certain limitations warrant discussion. First, this study assessed only acute (24-h) outcomes; long-term behavioral, cognitive, and regenerative effects require further exploration. Second, detailed pharmacokinetic studies measuring plasma and brain concentrations are needed to validate *in silico* predictions and quantify BBB penetration. Third, while the chosen dose range was based on prior evidence and demonstrated safety, more refined dose–response studies could optimize therapeutic efficacy. Fourth, our results demonstrate correlation between ER stress attenuation and neuroprotection, but causality remains to be established. Future studies should use genetic or pharmacological manipulation of ER stress pathways (e.g., CHOP silencing, GRP78 modulation) to confirm mechanistic specificity. Finally, formulation strategies such as nanoparticle or liposomal encapsulation could be explored to further enhance brain bioavailability and support potential clinical translation.

Collectively, these findings highlight hypericin as a multitarget neuroprotective agent capable of mitigating cerebral I/R injury by attenuating ER stress–mediated apoptosis, preserving neuronal structure, and maintaining functional recovery. The dual demonstration of efficacy and safety underscores its therapeutic promise for ischemic stroke, meriting further preclinical and pharmacological optimization.

## 5 Conclusion

In conclusion, this study demonstrates that hypericin exerts potent neuroprotective effects against cerebral ischemia/reperfusion injury by improving neurological function, reducing infarct volume, mitigating neuronal apoptosis, and suppressing endoplasmic reticulum stress–associated signaling pathways. These findings position hypericin as a promising therapeutic candidate for ischemic stroke. However, further investigations involving mechanistic validation, long-term behavioral and cognitive assessments, pharmacokinetic profiling, and comprehensive safety evaluations are essential to facilitate its potential clinical translation.

## Data Availability

The raw data supporting the conclusions of this article will be made available by the authors, without undue reservation.
