# Supplementary material for: Hypericin alleviates cerebral ischemia/reperfusion injury by modulating endoplasmic reticulum stress
Source: Front Pharmacol. 2026 Jan 7;16:1723495. doi: 10.3389/fphar.2025.1723495 (PMC12819193; doi:10.3389/fphar.2025.1723495)
Supplement: Supplementary file 1 [file DataSheet1.docx]

**Supplementary Data**

**Supplementary Table S1:** List of primer sequences used in the study

| Gene | Direction | Sequence |
| --- | --- | --- |
| Bax | Forward | AGGATGCGTCCACCAAGAAGCT |
|  | Reverse | TCCGTGTCCACGTCAGCAATCA |
| Bcl-2 | Forward | CCTGTGGATGACTGAGTACCTG |
|  | Reverse | AGCCAGGAGAAATCAAACAGAGG |
| Caspase-3 | Forward | GGAGTCTGACTGGAAAGCCGAA |
|  | Reverse | CTTCTGGCAAGCCATCTCCTCA |
| Caspase-9 | Forward | GCTGTGTCAAGTTTGCCTACCC |
|  | Reverse | CCAGAATGCCATCCAAGGTCTC |
| Caspase-12 | Forward | TCCAACGGTGTTCTGGTCC |
|  | Reverse | TCTCGCATCCCCAAAAGGTC |
| CHOP | Forward | TGTTGAAGATGAGCGGGTGG |
|  | Reverse | GATGGTGCTGGGTACACTT |
| GRP78 | Forward | GTGTGTGAGACCAGAACCGT- |
|  | Reverse | TCGCTGGGCATCATTGAAGT |

**Supplementary Table S2.** In-silico physicochemical and pharmacokinetic properties of Hypericin (SwissADME analysis)

| Category | Parameter | Result / Value | Interpretation / Implication |
| --- | --- | --- | --- |
| Physicochemical properties | Molecular formula | C₃₀H₁₆O₈ | Polycyclic aromatic compound |
|  | Molecular weight | 504.44 g/mol | Slightly above ideal oral range (violates Lipinski MW > 500) |
|  | Molar refractivity | 142.91 Å³ | Consistent with aromatic planar structure |
|  | Topological polar surface area (TPSA) | 155.52 Å² | High polarity limits passive diffusion and BBB permeability |
|  | H-bond donors / acceptors | 6 / 8 | Excess donors may hinder absorption |
|  | Rotatable bonds | 0 | Highly rigid aromatic skeleton |
| Lipophilicity | Consensus Log P | 4.53 | Highly lipophilic; favors membrane association |
|  | Range (iLOGP – SILICOS-IT) | 3.13 – 6.90 | Confirms strong lipophilic nature |
| Water solubility | Log S (ESOL) | –7.82 (7.6 × 10⁻⁶ mg/mL) | Poorly soluble in aqueous medium |
|  | Log S (Ali / SILICOS-IT) | –9.98 / –8.24 | Very low solubility; requires DMSO or surfactant-based vehicle |
| Pharmacokinetics | GI absorption | Low | Predicts limited oral absorption |
|  | BBB permeant | No | Poor predicted BBB penetration under normal physiological conditions |
|  | P-gp substrate | No | Not effluxed by P-glycoprotein |
|  | CYP inhibition profile | Inhibits CYP2C9 and CYP2C19 | Possible hepatic metabolism via these pathways |
|  | Log Kp (skin permeation) | –4.48 cm/s | Low dermal permeability |
| Drug-likeness | Lipinski criteria | 2 violations (MW > 500, H-don > 5) | Moderate deviation from ideal drug-like profile |
|  | Veber / Egan | 1 violation (TPSA > 140 Å²) | Suggests limited permeability |
|  | Bioavailability score | 0.17 | Low oral bioavailability |
| Medicinal chemistry | PAINS alert | 0 | No pan-assay interference risk |
|  | Brenk alert | 1 (polycyclic aromatic hydrocarbon) | Structural alert common for aromatic phytochemicals |
|  | Synthetic accessibility | 2.98 | Moderate synthetic feasibility |
| Overall interpretation | — | — | Poor aqueous solubility and limited BBB permeability; however, strong lipophilicity and neuroactive potential suggest suitability for intraperitoneal or nanoparticle-based CNS delivery. |

**
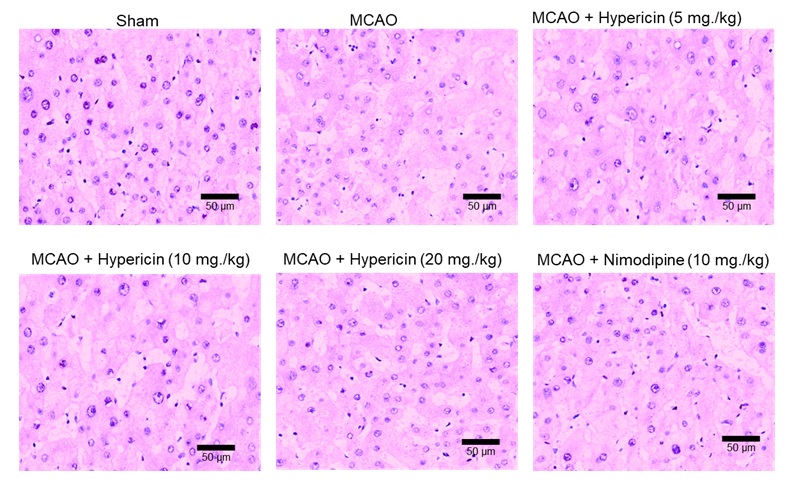
 Supplementary Figure S1. Histopathological evaluation of liver tissues following hypericin treatment in cerebral ischemia/reperfusion (I/R) rats.** Representative hematoxylin and eosin (H&E)–stained liver sections (magnification ×100; scale bar = 50 µm) are shown. The sham and MCAO groups exhibited normal hepatic architecture with hepatocyte cords radiating from the central vein, intact sinusoidal spaces, and preserved nuclear morphology. Similarly, livers from rats treated with hypericin (5, 10, and 20 mg/kg) or nimodipine (10 mg/kg) showed no evidence of hepatocellular necrosis, inflammatory infiltration, or cytoplasmic degeneration. These observations confirm the absence of hepatotoxic effects at the administered doses of hypericin.
